# Optimizing Nitrate and Tryptone to Enhance Growth and Triacylglycerol Accumulation in *Phaeodactylum tricornutum*

**DOI:** 10.4014/jmb.2408.08036

**Published:** 2024-09-30

**Authors:** Yufang Pan, Zhaowen Hu, Eric Maréchal, Hanhua Hu

**Affiliations:** 1 Key Laboratory of Algal Biology, Institute of Hydrobiology, Chinese Academy of Sciences, Wuhan 430072, P.R. China; 2 University of Chinese Academy of Sciences, Beijing 100049, P.R. China; 3 Laboratoire de Physiologie Cellulaire et Végétale, Institut National de Recherche pour l'Agriculture, l'Alimentation et l'Environnement, Centre National de la Recherche Scientifique, Commissariat à l’Energie Atomique et aux Energies Alternatives, Université Grenoble Alpes; IRIG, CEA-Grenoble, 17 rue des Martyrs; 38000 Grenoble, France

**Keywords:** Amino acids, culture medium, *Phaeodactylum tricornutum*, triacylglycerol, tryptone

## Abstract

*Phaeodactylum tricornutum*, a unicellular diatom, is considered a potential feedstock for the production of biofuel and a promising producer for high-value products eicosapentaenoic acid and fucoxanthin. However, a high-efficient cultivating strategy to achieve commercial production of triacylglycerol (TAG) from the diatom is an urgent demand. In this study, we optimized the content and ratio of nitrate and tryptone in the medium to enhance biomass and TAG accumulation simultaneously. Growth with tryptone as the sole nitrogen gave rise to the lowest cell density but the highest TAG content in *P. tricornutum* relative to nitrate, nitrite, ammonium or urea cultures. In 500 μM NaNO_3_ cultures, the growth of *P. tricornutum* increased with the increasing concentration (from 294 to 7056 μM nitrogen) of supplemented tryptone, however supplementation of high tryptone (≥882 μM nitrogen) decreased the neutral lipid content. Elevating nitrogen concentration from 294 to 882 μM via tryptone addition in 250 μM nitrate culture increased cell densities from day 6 to 10 and neutral lipid content on day 10. In particular, supplementing 588 μM nitrogen of tryptone in the 250 μM nitrate culture gave rise to the highest neutral lipid content on days 8 and 10 (increased by 109% and 62% relative to 500 μM nitrate-sole) with a comparable growth to that in 500 μM nitrate-sole culture from day 2 to 8. In conclusion, we optimized nitrate/tryptone ratio and found that a suitable tryptone addition to a relatively low nitrate culture was favourable to the biomass and TAG accumulation simultaneously in *P. tricornutum*.

## Introduction

*Phaeodactylum tricornutum*, a unicellular marine pennate diatom, is a common experimental model system for diatom biology study [1]. It is also considered as a potential feedstock for the production of biofuel because of its relatively high triacylglycerol (TAG) productivity [2], and its fatty acid composition meets the requirements of international biodiesel standards [3]. *P. tricornutum* has been shown to dominate and outcompete other microalgae species in outdoor mass-culture systems due to its tolerance to high pH, and growth ability under low light [4, 5]. In addition, *P. tricornutum* is a promising producer for high-value products such as eicosapentaenoic acid (EPA), chrysolaminarin, and fucoxanthin, and can serve as a suitable microalgal cell factory [6, 7].

Nitrogen is the indispensable nutrient in the biosynthesis of chlorophylls and proteins. *P. tricornutum* can utilize not only inorganic nitrogen sources like nitrate, nitrite and ammonium but also organic nitrogen sources like urea, tryptone and most of the amino acids [8-10]. *P. tricornutum* was shown to acclimate to environments with variable nitrogen sources by specific signaling pathways. For instance, when *P. tricornutum* is transferred from a high nitrate and low nitrite medium to a low nitrate and high nitrite one, nitrite serves as a substrate for nitric oxide (NO) production by nitrate reductase; this labile gaseous signal then triggers the expression of nitrite reductase to cope with the new condition [11]. This diatom is a successful photoautotroph but is also capable of mixotrophic growth on organic carbon sources like glucose, acetate, fructose and glycerol [12-16]. Conflicting lines of evidence showed that *P. tricornutum* could not natively consume glucose but can heterotrophically grow with glucose by introducing the human glucose transporter gene *Glut1* [17] or the *Chlorella kessleri* hexose uptake protein (HUP1) gene [18]. Numerous researchers have been devoted to the studies of biomass and high-value product improvement in *P. tricornutum* by medium optimization [9, 19-21].

Tryptone provides a complex nutrition (free amino acids, peptides, vitamins and growth factors) and has been used for the cultivation of *P. tricornutum* as early as 1958 [22]. Compared to the conventional nitrogen source, sodium nitrate (NaNO_3_), the mixture of tryptone and urea as the organic nitrogen source can induce a higher biomass and fucoxanthin production in *P. tricornutum* [21]. Enriching the f/2 medium with tryptone and yeast extract increases biomass, fucoxanthin production and TAG content up to 3.48-fold, 1.7-fold and 2.13-fold, respectively in *P. tricornutum* [23].

Neutral lipids (mainly TAGs) increase in nitrogen-deprived cultures while the photosynthetic capacity and chlorophyll content decrease in *P. tricornutum* [24], indicating that it is hard to accumulate biomass and TAGs simultaneously. The small (27 Mb) and well-characterized genome, available engineering tools, comprehensive knowledge of lipids biosynthetic pathways and their regulation, robust growth in mass-culture systems, tolerance of low-light levels and high pH suggest that *P. tricornutum* has great potentials of application [6]. Therefore, there is an urgent demand for the development of a high-efficient cultivating strategy to achieve commercial production of TAGs from the diatom. Glycerol and urea are often used as organic carbon and nitrogen sources, respectively, in mixotrophic growth of *P. tricornutum* [13-16], and tryptone containing both organic nitrogen and organic carbon has received little attention. In this study, we optimized the content of NaNO_3_ and tryptone in the medium, and the results could provide the basis for the mixotrophic cultivation strategy that significantly increases neutral lipid production without reducing biomass of *P. tricornutum*.

## Materials and Methods

### Strain and Growth Conditions

*P. tricornutum* (CCMP2561), a gift from Chris Bowler, originated from the Provasoli-Guillard National Center for Marine Algae and Microbiota (NCMA, USA). For growth experiments in different nitrogen sources, algal cells were cultivated in the 250-ml Erlenmeyer flask containing 120 ml artificial seawater enriched with N-free f/2 [25], and 500 μM nitrogen was added to the culture with NaNO_3_, NaNO_2_, NH_4_Cl, urea or tryptone as the sole nitrogen source. The cultures were incubated in a shaker-incubator at 130 rpm with the initial cell density of 2 × 10^5^ cells ml^-1^ under a continuous light irradiation of 70 μmol photons m^-2^ s^-1^ at 22°C. To eliminate the effect of intracellular nitrogen sources, nitrogen-starved cells [26] were used as the inoculum. For the medium optimization experiments, nitrate and tryptone of various concentrations were used as the nitrogen source, and algal cells were cultivated in the 150-ml Erlenmeyer flask containing 60 ml medium incubated in a shaker-incubator at 100 rpm. For the fatty acid analysis, 200 ml cultures were grown in the 500-ml Erlenmeyer flask shaken at 130 rpm or in the glass tubes (36-cm long and 3.5-cm diameter) bubbled with filtered air at 0.4 vvm (air volumes per volume of reactor per minute). For the growth experiment with amino acids, NaNO_3_ in f/2 was replaced with an individual out of the 20 amino acids at the nitrogen concentration of 882 μM, cultivation was performed in 50-ml Erlenmeyer flask containing 35 ml medium and shaken manually three times a day. For the growth analysis, 3 biological replication was conducted and cell numbers were counted using a Countstar automated algae counter (Countstar algae, China).

### Nitrogen Concentration Measurement

Concentrations of nitrate and nitrite in the cultures were measured according to Collos *et al*. [27]. In brief, the culture supernatant was collected and the absorbance was measured at 220 nm, and sodium nitrate at a concentration range of 0-882 μM was used as the standard. Ammonium nitrogen concentration was determined by standard Nessler's reagent spectrophotometric method. In details, 2 ml of culture was centrifuged at 10,000 rpm for 10 min, and then 0.5 ml of supernatant was added into a colorimetric tube and diluted to 5 ml with ultrapure water. 0.05 ml potassium sodium tartrate solution (500 g/l) was added to the colorimetric tube and mixed. 0.1 ml of Nessler's reagent was added to the tube and mixed. After 10 min, the absorbance of the solution was measured at a wavelength of 420 nm with ultrapure water as a reference. Concentration of urea was measured at the wavelength of 426 nm after chromogenic reaction of urea with 4-dimethylaminobenzaldehyde [28]. To be specific, a solution (2 ml) containing 2% (w/v) of 4-dimethylaminobenzaldehyde and 6% (v/v) hydrochloric acid in absolute ethanol was added to 3 ml of the supernatant. After 20 min, the absorbance of the solution was measured at 426 nm.

### Relative Quantification of Triacylglycerol by Thin-Layer Chromatography and Nile Red Staining

Total lipids were extracted from dry algal biomass using chloroform/methanol (1:1, v/v) [29], and then were developed using one-dimensional thin-layer chromatography (TLC) in hexane:diethyl ether:acetic acid (70:30:1, v/v/v) on silica gel plates 60 F254 (Merck KgaA, Germany). TAG bands on the plates were identified by staining with iodine, and triolein was used as the standard (Sigma-Aldrich). TAG relative abundance was subjected to densitometric scanning using Image J software. Relative abundance of the neutral lipid (mainly TAGs) was estimated by fluorescence spectroscopy (Perkin-Elmer, LS 55) with the fluorescent dye Nile red (Sigma-Aldrich) according to procedures described before [30]. Briefly, equal numbers of cells (6 × 10^6^ cells) were harvested and resuspended to 3 ml, and then 30 μl (100 μg/ml) Nile red staining solution was added at 37°C for 30 min and subsequently excited at 531 nm, and the fluorescent emission was measured at 572 nm. Background fluorescence for this filter set was subtracted prior to the addition of Nile red.

### Fatty Acid and Free Amino Acid Analysis

TAGs fractionated from the TLC plate and dry algal cells were esterified directly by heating in 5% sulfuric acid in methanol. Then the fatty acid methyl esters were analyzed by gas chromatography (GC9790II, Fuli instruments, China) as reported before [31].

Free amino acids in the sterilized tryptone medium were analyzed using A300 amino acid analyzer (membraPure Bodenheim, Germany) with the ion-exchange chromatographic column (650-0042, membraPure Bodenheim, Germany). After reacting with ninhydrin (20 g/l) for an hour, the resultant derivatives were measured by UV detection at wavelengths of 440 and 570 nm, and the derivative of proline (Pro) was only detected at 440 nm [32].

## Results and Discussion

### Nitrogen Source Affects Growth and TAG Accumulation

**Fig. 1:**
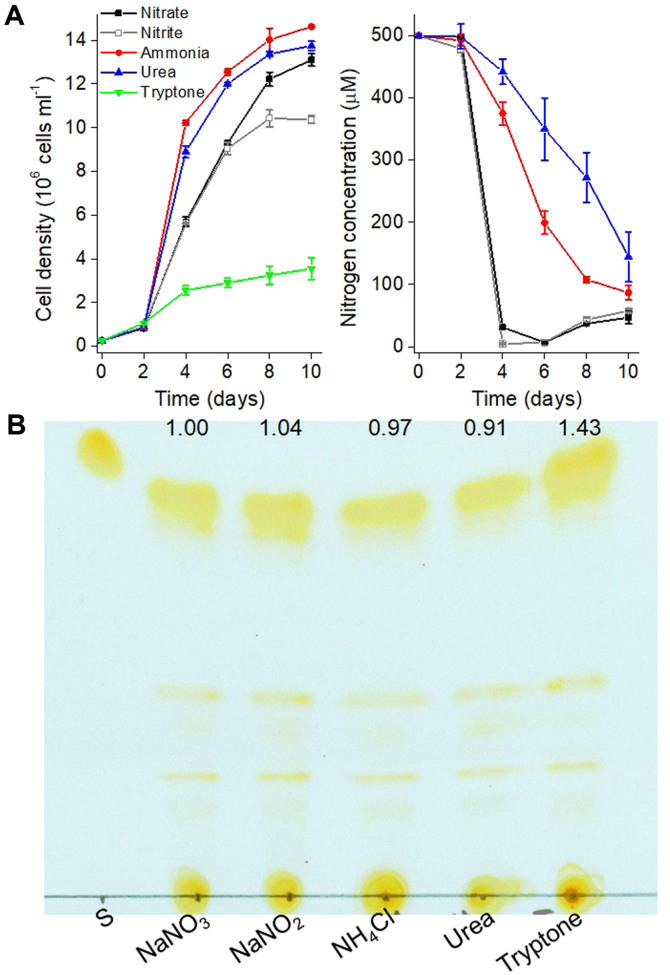
Effect of nitrogen sources on *Phaeodactylum tricornutum*. (**A**) Growth and nitrogen utilization of algal cells grown in f/2 enriched artificial seawater medium (nitrogen concentration was set at 500 μM with NaNO_3_, NaNO_2_, NH_4_Cl, urea, or tryptone as the sole nitrogen sources); (**B**) TAG content analyzed by thin-layer chromatography in algal cells grown on day 10. S: 0.02 mg triolein as the standard for triacylglycerol.

*P. tricornutum* can utilize both inorganic (nitrate, nitrite, and ammonium) and organic nitrogen (urea and amino acids) as the sole nitrogen sources [10, 33]. Inorganic nitrogen incorporation operates via stepwise reductions, i.e. nitrate (by nitrate reductase) to nitrite (by nitrite reductase) and to ammonium. Ammonium is then the entry point in *P. tricornutum* intracellular carbon metabolism via two competing routes: on the one hand the glutamine synthase (GS)/glutamate synthase (GOGAT) route, and on the other hand the carbamoyl-phosphate route producing arginine (Arg) [11]. In this study, five nitrogen sources were added individually to the medium to obtain a concentration of 500 μmol of nitrogen per liter. During the 10-day cultivation period (Fig. 1A), ammonium chloride gave rise to the highest cell density of 1.47 × 10^7^ cells ml^-1^, consistent with the role of this reduced inorganic nitrogen source as the entry point in intracellular carbon metabolism, and the cell densities were 10% and 6% lower when nitrate and urea was used respectively as the nitrogen source. Although the growth was not different from day 0 to day 6 with nitrate and nitrite as the sole nitrogen source, the cell density decreased by 15% and 21% respectively on days 8 and 10 under nitrite condition relative to nitrate condition. Contreras and Gillard show that the growth seems to be almost the same in *P. tricornutum* grown with 200 μM NH_4_Cl or NaNO_3_ as the sole nitrogen source before day 14 [10]. According to Fidalgo *et al*. [33], growth with 4 mM nitrogen of NaNO_3_, NaNO_2_ and urea was not much different before day 8 while the highest cell density of 0.88 × 10^7^ cells ml^-1^ was achieved on day 11 in nitrate cultures; however, the growth of *P. tricornutum* cultured with 4 mM nitrogen of ammonia ceased on day 4. Collos and Harrison indicate that the mean optimal ammonium concentration was 340 μM and the tolerance to high toxic ammonium level was 3,600 μM in diatoms [34]. In our study, cultivation was performed at 500 μM ammonium with the initial pH 8.0, and the highest cell density was observed in cultures with ammonium as the sole nitrogen source, indicating that even if pH values changed, no negative effect on the growth was noticed. Furthermore, pH values only change slightly based on the findings of Collos and Harrison [34] at 500 μM ammonium condition.

Supplementing tryptone in the medium containing urea or nitrate enhances the growth of *P. tricornutum* [21, 23], while the effect of tryptone as the sole nitrogen on its growth is unknown. We found the growth of *P. tricornutum* was the slowest in medium with tryptone as the sole nitrogen and the lowest cell density of 3.6 × 10^6^ cells ml^-1^ was observed on day 10. The absorption of nitrate and nitrite was fast in the culture of *P. tricornutum*, and they were almost exhausted on day 4, while nitrogen concentration maintained a level of about 100 μM in the culture with urea or ammonium as the sole nitrogen source on day 10. This supports that, although ammonium is the key entry in intracellular carbon metabolism, nitrate and nitrite import were the most efficient. Cell density decrease observed on day 8 and 10 with nitrite instead of nitrate further supports that *P. tricornutum* nitrogen incorporation is best adapted to a natural supply of nitrate. TLC analysis showed a 43%, 38%, 47%, and 52%increase of TAG content in *P. tricornutum* grown with tryptone compared those with nitrate, nitrite, ammonium, and urea on day 10, respectively (Fig. 1B).

### Optimizing Tryptone Concentration in the Medium with Nitrate

**Fig. 2:**
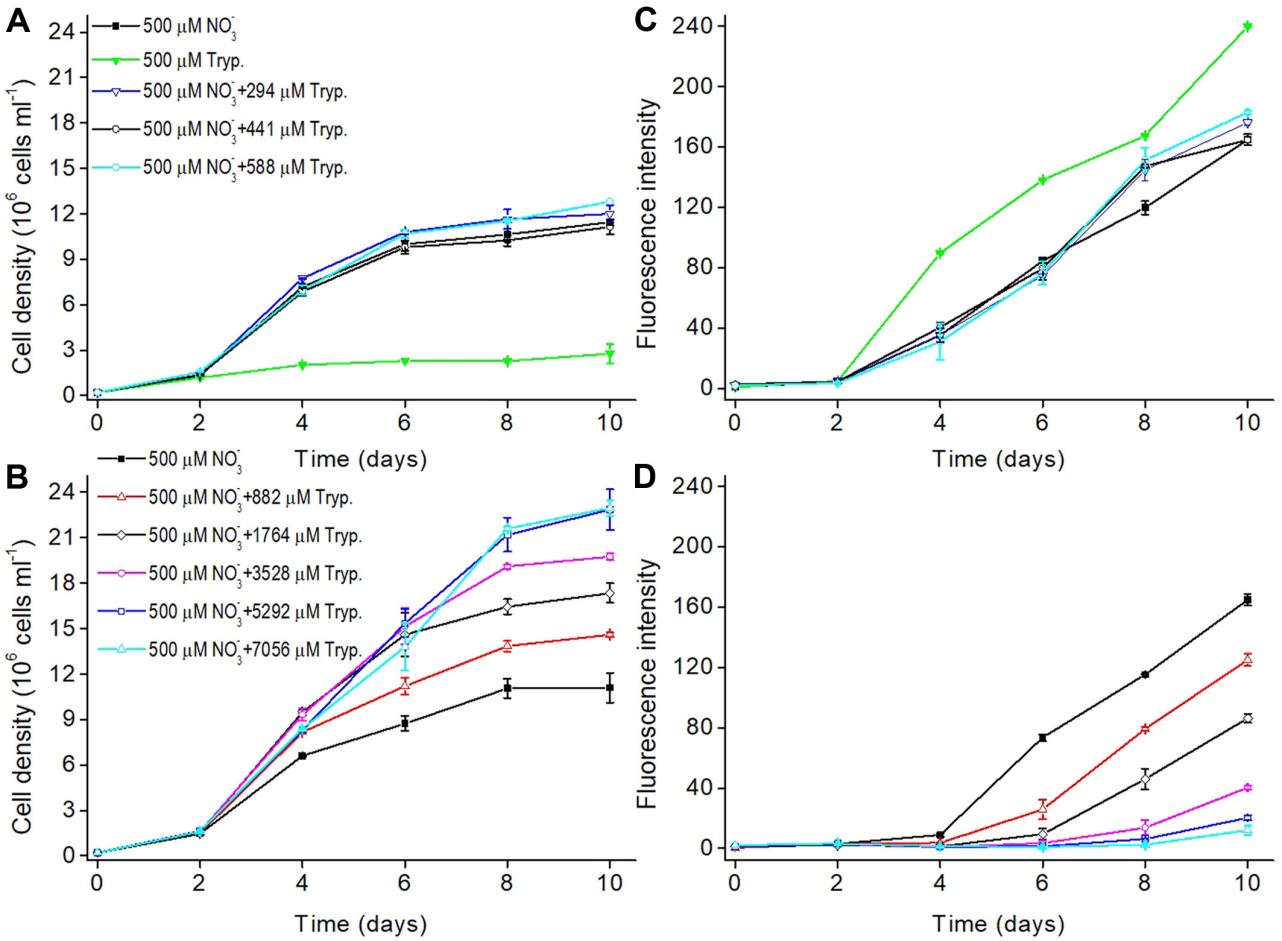
Optimizing tryptone concentration at 500 μM NaNO_3_ for *Phaeodactylum tricornutum*. (**A**) Growth curves of algal cells in the medium with tryptone of low concentration (≤588 μM nitrogen of tryptone); (**B**) Growth curves of algal cells in the medium with tryptone of high concentration (≥882 μM nitrogen of tryptone); (**C**) Accumulation of neutral lipid (fluorescence intensity normalized to cell number) detected by Nile red assay at low concentration tryptone; (**D**) Accumulation of neutral lipid at high concentration tryptone.

Although cultivation with tryptone alone resulted in the highest TAG content, the cell density was only about 1/ 4 of that in cultivation with nitrate, indicating that tryptone as a single nitrogen source is not suitable for the cultivation of *P. tricornutum*. To obtain the optimal ratio of inorganic and organic nitrogen sources, different ratios (ranging from 0.07 to 2.33) of NaNO_3_ and tryptone were used to cultivate the diatom. In 500 μM NaNO_3_ cultures, supplementing more tryptone (from 294 to 7056 μM nitrogen) enhanced the growth of *P. tricornutum*, and cell density on day 10 increased by 8%, 15%, 32%, 56%, 78%, 106%, and 107% in 294, 588, 882, 1764, 3528, 5292, and 7056 μM nitrogen of tryptone (namely 1/3, 2/3, 1, 2, 4, 6, and 8-fold of 882 μM nitrogen of tryptone respectively) relative to 500 μM NaNO_3_ alone (Fig. 2A and 2B). In addition, supplementing 294 and 588 μM nitrogen of tryptone increased the neutral lipid content by 7% and 11%, however supplementation of higher tryptone decreased the neutral lipid content by 32%, 56%, 78%, 106%, and 107% respectively in 882, 1764, 3528, 5292, and 7056 μM nitrogen of tryptone relative to 500 μM nitrate cultures (Fig. 2C and 2D). Furthermore, the neutral lipid content was still lower in 294, 441 and 588 μM nitrogen of tryptone cultures when compared with 500 μM nitrogen of tryptone culture alone (Fig. 2C), which indicated that supplemented nitrogen of tryptone should not beyond 882 μM considering the TAG content.

**Fig. 3:**
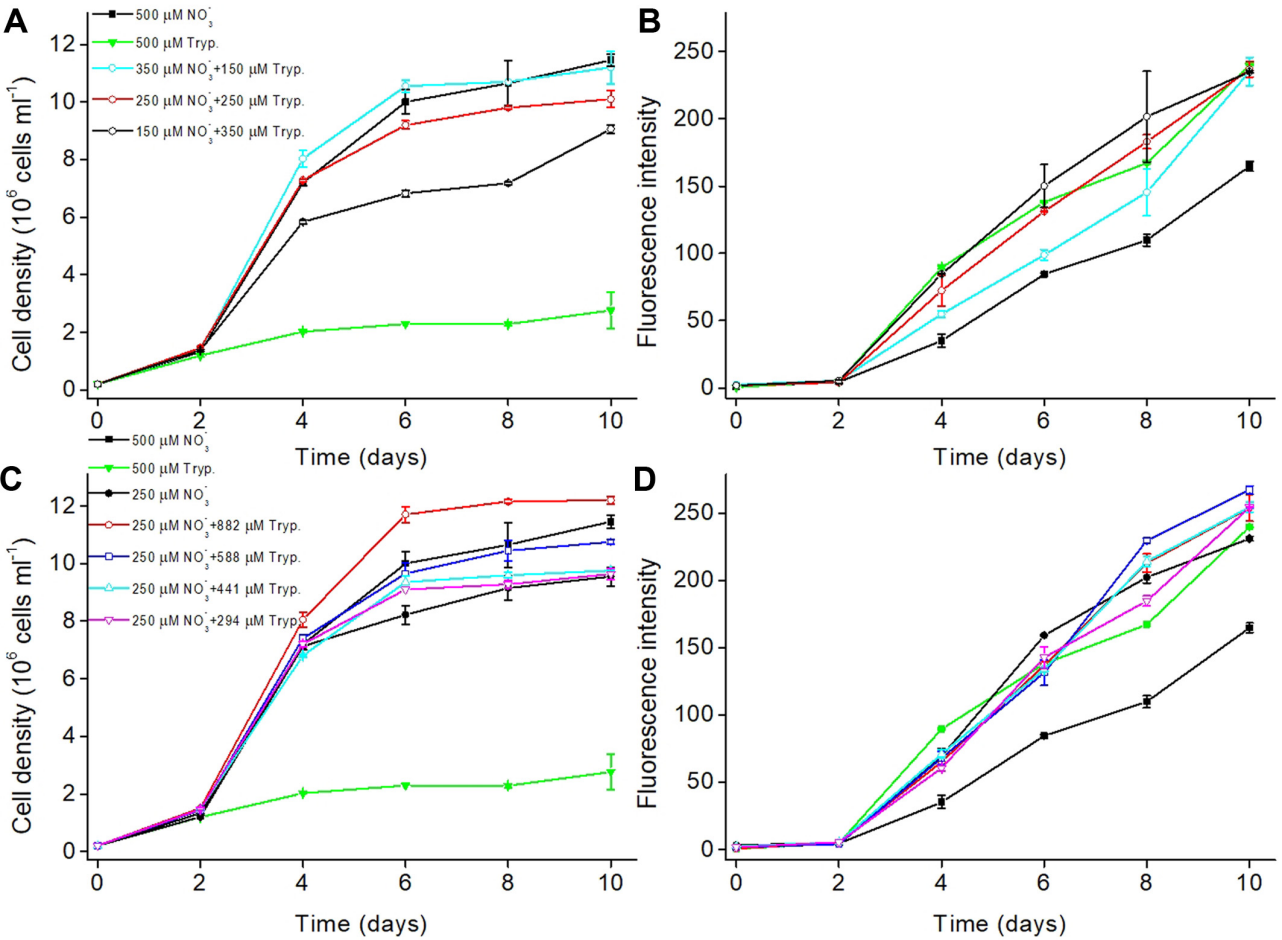
Optimizing tryptone concentration at low NaNO_3_ for *Phaeodactylum tricornutum*. (**A**) Growth curves of algal cells in the medium containing 500 μM nitrogen in total; (**B**) Growth curves of algal cells in the medium containing 250 μM NaNO_3_ supplemented with tryptone of different concentrations; (**C**) Accumulation of neutral lipid (fluorescence intensity normalized to cell number) detected by Nile red assay at 500 μM nitrogen in total; (**D**) Accumulation of neutral lipid at 250 μM NaNO_3_ supplemented with tryptone of different concentrations.

Since supplementing 294-588 μM nitrogen of tryptone only slightly enhanced the growth as well as TAG content in *P. tricornutum* (Fig. 2A and 2C) and further elevating tryptone increased biomass with a huge sacrifice of TAG accumulation, it seemed that a total of about 500 μM nitrogen might be suitable for the 10 days’ cultivation. Therefore, further experiments were carried out for the optimization of ratio between nitrate and tryptone with a total of 500 μM nitrogen. Adding 150 μM nitrogen of tryptone to the culture with 350 μM nitrate brought about the growth comparable to that in cultures with 500 μM nitrate (Fig. 3A), while increasing the neutral lipid content by 56%, 17%, 32%, and 43% respectively on day 4, 6, 8, and 10 (Fig. 3B). Supplementing 250 μM nitrogen of tryptone to the culture with 250 μM nitrate decreased the cell densities by 8%, 8%, and 12% respectively on day 6, 8, and 10 but increased the neutral lipid content by 106%, 55%, 67%, and 44% respectively on day 4, 6, 8, and 10 (Fig. 3B). Although cultivation with 150 μM nitrate and 350 μM nitrogen of tryptone gave rise to the highest neutral lipid content on day 6 and 8, cell density was much lower from day 4 to 10 compared with the nitrate culture and the other two supplemented cultures (Fig. 3A and 3B).

Based on the further optimization of the ratio between nitrate and tryptone, supplementing 250 μM nitrogen of tryptone to the culture with 250 μM nitrate could accumulate more neutral lipids without intense inhibition of growth. Thus, 250 μM nitrate was used in the subsequent study. Elevating the concentration of nitrogen of tryptone from 294 to 882 μM in the culture with 250 μM nitrate increased cell densities from 9.6 to 12.2 × 10^7^ cells ml^-1^ on day 10. To be specific, on day 10 cell density was 1%, 2%, 13%, and 28% higher respectively in 294, 441, 588, and 882 μM nitrogen of tryptone-supplemented cultures relative to 250 μM nitrate culture alone (Fig. 3C). In particular, supplementing 588 μM nitrogen of tryptone in the 250 μM nitrate culture even gave rise to the highest neutral lipid content on days 8 and 10 (increased by 109% and 62% relative to 500 μM nitrate) (Fig. 3D) with a comparable growth to that in 500 μM nitrate-sole culture from day 2 to 8 (Fig. 3C). Lower nitrate in the medium resulted in higher neutral lipid accumulation in the culture of *P. tricornutum* regardless of tryptone (no more than 882 μM nitrogen) addition (Fig. 3B and 3D), however supplementation of tryptone could enhance the neutral lipid accumulation at the end of cultivation. The previous study has reported that supplementation of 1 g l-1 tryptone (equivalent to 9 mM nitrogen), much higher than that used in our study, to f/2 medium containing 882 μM nitrate significantly increased cell growth, TAG and fucoxanthin content in *P. tricornutum* [23]. Our present study optimized the ratio of nitrate and tryptone and found that a suitable tryptone addition to a relatively low nitrate culture was favorable to the biomass and TAG accumulation simultaneously.

### Fatty Acid Composition at the Optimizing Tryptone Concentration

**Fig. 4:**
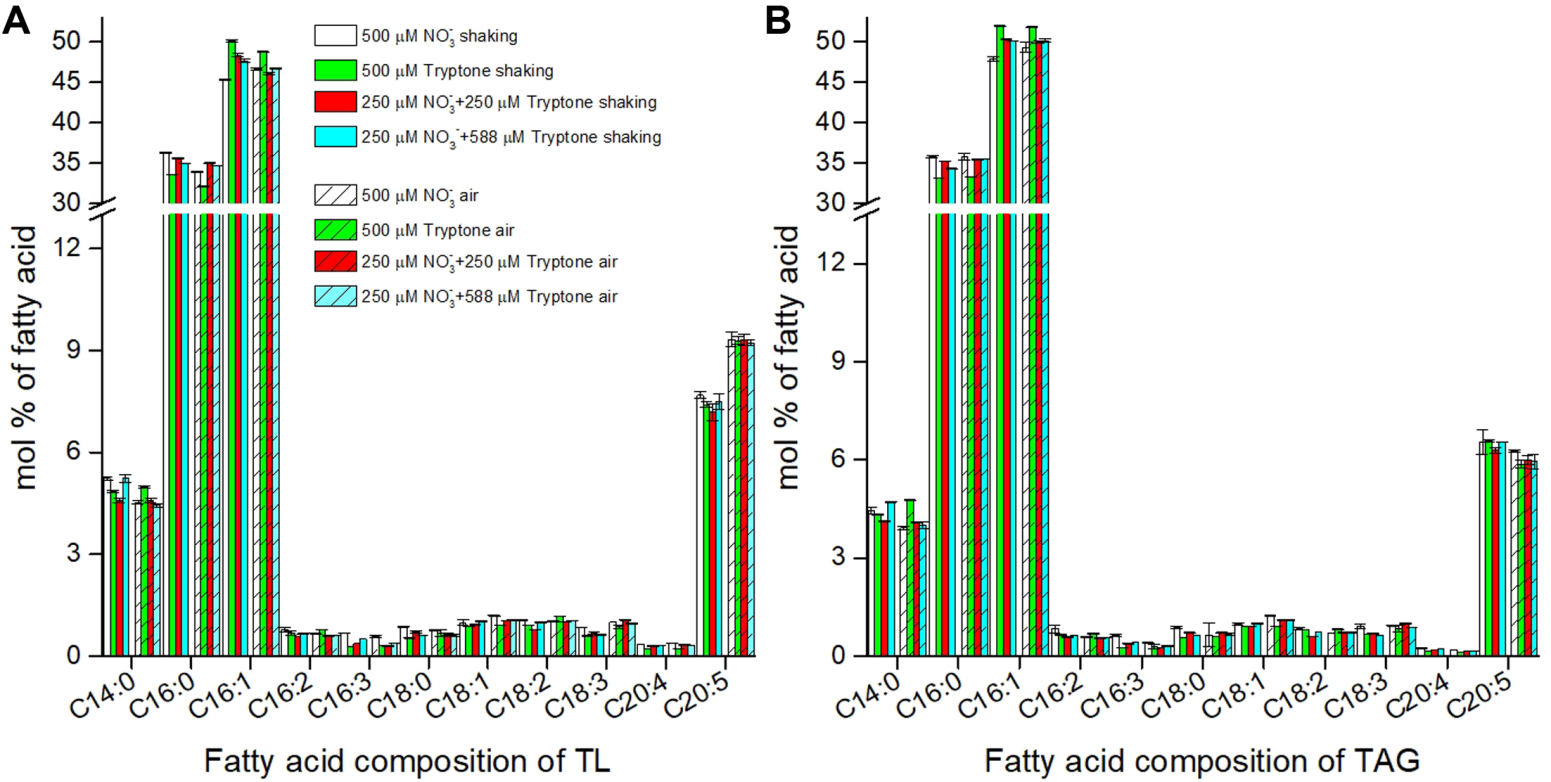
Comparative analysis of fatty acid composition of *Phaeodactylum tricornutum* grown in the optimized medium in a shaker-incubator (shaking) or in the glass tubes bubbled with air (air). (**A**) Fatty acid composition in total lipid (TL); (**B**) Fatty acid composition in triacylglycerol (TAG).

To investigate whether tryptone supplementation has an impact on fatty acid composition, algal cells were grown for 7 days (aeration cultures) or 10 days (shaking cultures) in 500 μM nitrate-sole, 500 μM nitrogen of tryptone-sole, and 250 μM nitrate supplemented with 250 μM or 588 μM nitrogen of tryptone cultures, and fatty acids were analyzed by GC. Nitrogen sources did not much affect the fatty acid composition and content, and the main fatty acids C14:0, C16:0, C16:1 and EPA accounted for 94.4-95.9% and 94.7-96.0% of the total fatty acids respectively in total lipid (Fig. 4A) and TAG (Fig. 4B). Likewise, when the diatom *Nitzschia laevis* was cultured with 7.3 mM nitrate supplemented with 4.5-36 mM nitrogen of tryptone, the EPA contents were similar to that of the control (7.3 mM nitrate-sole) [35]. However, cells with aeration gave rise to about 21-30% higher EPA in total lipid and slightly decreased EPA in TAG than shaking conditions (Fig. 4A), indicating that the increased EPA was mainly in polar lipids rather than in TAG. Elevation of CO_2_ has been shown to raise EPA content in *Nannochloropsis* sp. [36]. Maximum EPA production was observed in *P. tricornutum* when the air was supplemented with 1% CO_2_ [37]. Apparently, increased supply of CO_2_ is favorable to EPA accumulation.

### Effects of Sole Amino Acid on Growth and TAG Content

**Fig. 5:**
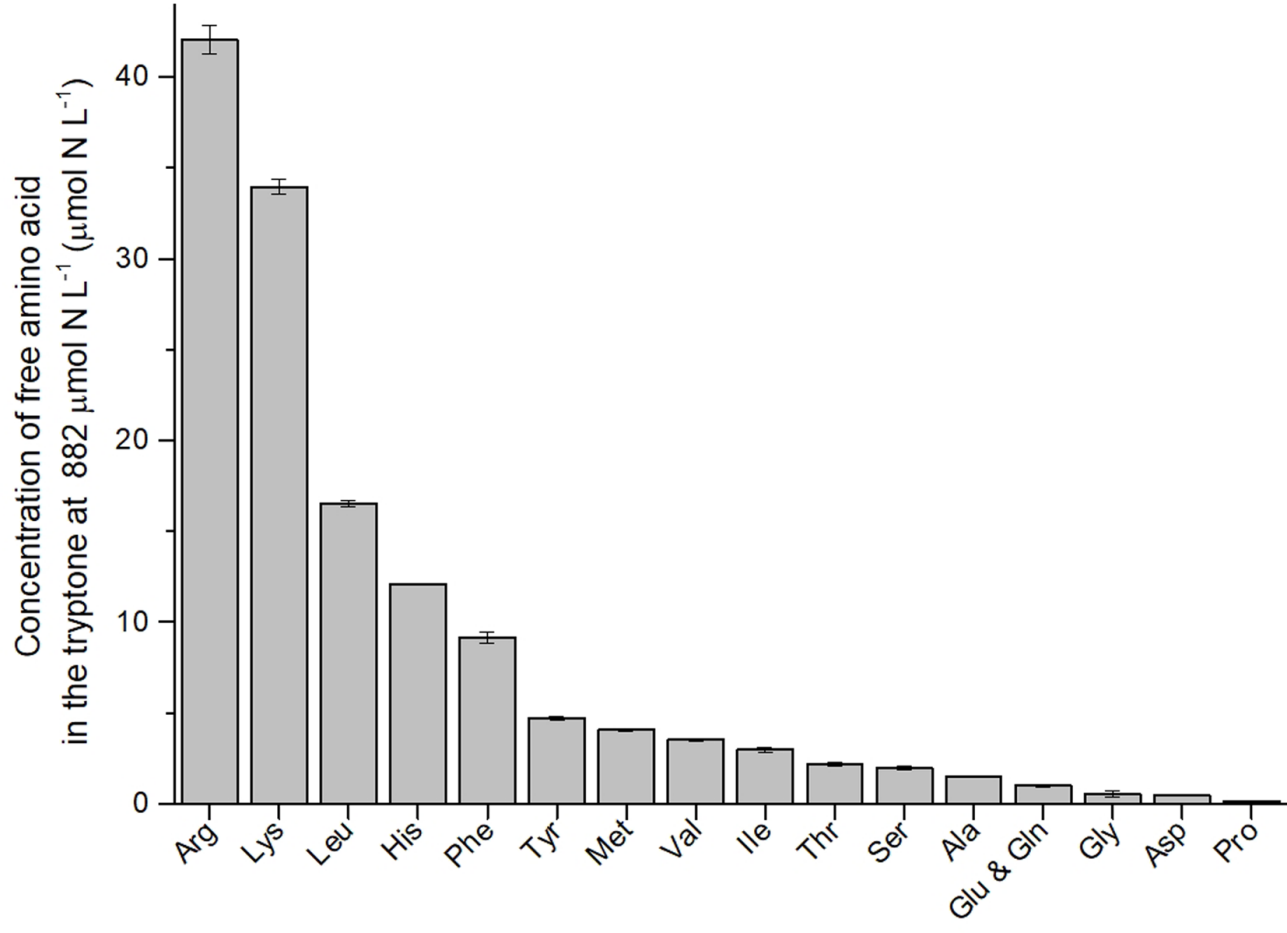
Concentration of free amino acids in the sterilized medium supplemented with 882 μM nitrogen of tryptone.

Tryptone is a pancreatic digest of casein containing all amino acids found in casein as well as larger peptide fractions. As a conventional source of nitrogen in medium for bacteria and fungi, tryptone contributes amino acids, nitrogen and vitamins. Utilizing different amino acids as nitrogen sources has different effects on growth of *P. tricornutum* [10] and thus TAG content probably. Free amino acid composition in the autoclaved medium containing 882 μM nitrogen of tryptone was detected by amino acid analyzer (Fig. 5). The total nitrogen concentration of free amino acids detected was 136.7 μM, and Arg, lysine (Lys), leucine (Leu) and histidine (His) were the four most abundant free amino acids in tryptone medium with the nitrogen concentration of 42.1, 33.9, 16.5 and 12.1 μM respectively. Contreras and Gillard [10] showed that growth of *P. tricornutum* with Arg, glutamate (Glu), Glutamine (Gln), Leu, isoleucine (Ile), and asparagine (Asn) as the sole nitrogen source (200 μM) exhibited comparable growth with that in nitrate cultures before day 18-19, and then strong negative effects of Asn were identified after day 19. The high impact on growth obtained with Gln and Glu is consistent with the early incorporation of inorganic ammonium in carbon metabolism via the GS/GOGAT route, whereas the impact of Arg relates to the early incorporation of ammonium via the carbamoyl-phosphate route [11].

**Fig. 6:**
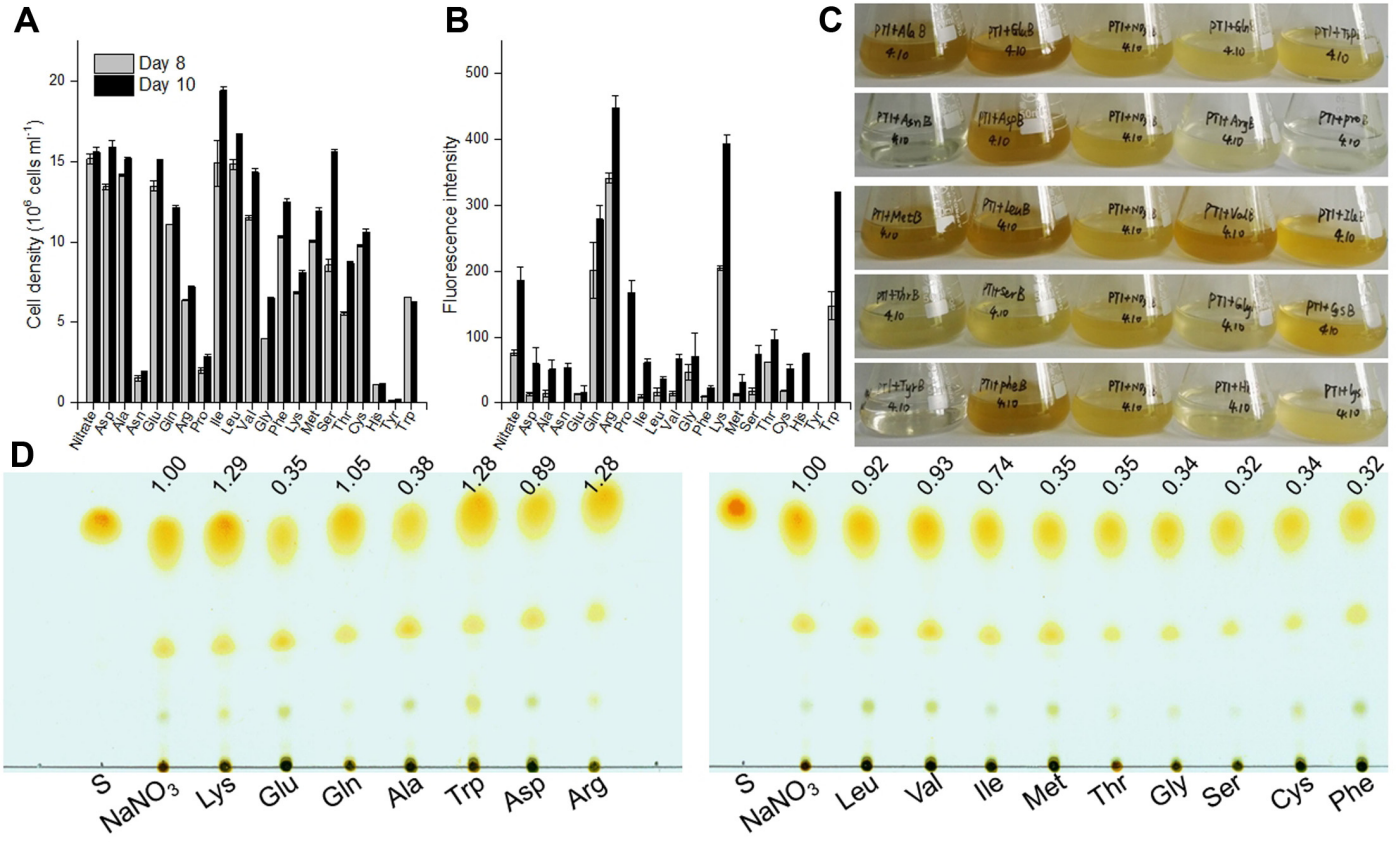
Effects of single amino acids as the sole nitrogen sources on *Phaeodactylum tricornutum*. (**A**) Cell density of algal cells grown in f/2 (nitrogen concentration was set at 882 μM with different amino acids) enriched artificial seawater medium on days 8 and 10; (**B**) Neutral lipid detected by Nile red assay on days 8 and 10; (**C**) Culture flasks at the end of the 10th day of growth; (**D**) TAG content analyzed by thin-layer chromatography in algal cells grown on day 10. S: 0.02 mg triolein as the standard for triacylglycerol.

To evaluate the impact of amino acids in tryptone on *P. tricornutum*, the diatom was cultured in a medium with all the 20 individual amino acids as the sole nitrogen source (882 μM nitrogen). Except that *P. tricornutum* grew very slowly with Asn, His and Pro and even could not survive with tyrosine (Tyr), its cell density was comparable in aspartate (Asp), alanine (Ala), Glu, valine (Val) and serine (Ser) with that in nitrate, and was lower in Gln, Arg, glycine (Gly), phenylalanine (Phe), Lys, methionine (Met), threonine (Thr), cysteine (Cys) and tryptophan (Trp), but higher in Ile and Leu at least on day 10 (Fig. 6A). Compared with the nitrate culture, more neutral lipids were detected by Nile red staining when the diatom was cultured with Arg, Lys, Gln or Trp as the sole nitrogen source (Fig. 6B), and accordingly these cultures were in a lighter brown color relative to the dark brown in the cultures of Asp, Ala, Glu, Val, Leu, Phe, and Met (Fig. 6C). It has been widely reported that the nitrogen limited cultures accumulate neutral lipids in *P. tricornutum* and the color of cultures was noticeably lighter [38, 39], as the chlorophyll a and fucoxanthin content decreased under nitrogen limitation [40, 41]. Likewise, in this study, we find the higher content of neutral lipids (detected by the Nile red staining fluorescence intensity) in cultures in a lighter brown. Interestingly, it was previously shown that nitrogen incorporation via the combined GS/GOGAT and carbamoyl-phosphate routes not only lead to Gln and Arg, but also to fumarate as a byproduct, feeding TAG production [11]. TLC analysis verified that growth with Lys, Trp, Arg and Gln produced more TAG in *P. tricornutum* (Fig. 6D).

Based on the results of amino acid composition and effects of individual amino acids on *P. tricornutum*, the promotion of growth by supplementing with tryptone to the medium might come from the additional nitrogen source provided by amino acids. However, at the same nitrogen concentration, growth with tryptone was worse than that with nitrate due to insufficient available nitrogen sources in tryptone. For example, supplementing 882, 588, 441 and 294 μM nitrogen of tryptone to the 250 μM nitrate cultures achieved the available nitrogen of 387, 341, 318 and 296 μM if only the free amino acids in tryptone was considered. This can explain why supplementing 441 and 294 μM nitrogen of tryptone resulted in a final cell density equivalent to that in 250 μM nitrate cultures, but much lower than that in 500 μM nitrate cultures. In addition, as we know, during the photosynthesis, the linear electron flow generates ATP and NADPH at a ratio of about 1.3, whereas the Calvin-Benson-Bassham (CBB) cycle requires the ATP and NADPH ratio of 1.5, resulting in a deficiency of ATP or an excess of NADPH [42-44]. Nitrate reduction to ammonia is accompanied by eight electron transfers resulting in the consumption of NAD(P)H. Therefore, relative to the organic nitrogen, inorganic nitrogen nitrate as nitrogen source could consume the excessive NAD(P)H, and thus enhance the photosynthesis.

## Conclusion

Although tryptone can serve as the sole nitrogen source for *P. tricornutum*, it should be of high concentrations because the available nitrogen concentration in the medium is too low. Furthermore, ammonium is the entry point to carbon metabolism, while nitrite and nitrate are incorporated with higher efficiency, nitrate being the more efficient inorganic nitrogen source. In addition, the use of nitrate can consume the excessive NAD(P)H and thus balance the ATP/NADPH ratio to meet the CBB cycle needs. In this study, we optimized nitrate/tryptone ratio and found that a suitable tryptone addition to a relatively low nitrate culture was favourable to the biomass and TAG accumulation simultaneously in *P. tricornutum*. Tryptone is one of the most commonly used organic nitrogen sources in the laboratory, however, its high cost and lower bioavailability limit the industrial-scale application. Organic wastewater contains components similar to tryptone and the optimization strategy based on this study provides potential application prospects for wastewater treatment.
